# Quality, Not Quantity, in Medical Education

**Published:** 1911-09

**Authors:** 


### Quality, Not Quantity, In Medical Education

AT the spring meeting of the Medical Sociey of the County of Erie, the Medical Department of the University of Buffalo initiated a movement, involving serial action by the State Society, the Regents and the Legislature, looking toward the requirement of a year in college, preparatory to the medical course. For a college, practically unendowed and limited by natural competitive factors to a comparatively small potential clientele, such an action betokens a high degree of moral courage and of self sacrifice.

Such action, however, is in line with the elevation of medical educational standards. In 1904, the number of medical schools had reached the maximum of 166. It is now 120 and must inevitably fall to 100 within a very few years. Forty-one schools already require at least one year of collegiate study and six more definitely announce such a requirement next year.

Those who have grown up with the idea that culture in America hugs the Atlantic shore and that the west is "wooly" will experience many shocks in studying educational statistics in general. There is, on the whole, less illiteracy and a greater proportion of high school graduates in the middle, northern states, than in any other part of the country. So far as standards for medical matriculants are concerned, the east can boast only of the commencement of the campaign by Johns Hopkins in 1893, and the falling into line of Harvard in 1900. Thereafter, most of the glory attaches to western standards (no pun intended) and the tardy enlistment of some of the oldest and strongest eastern schools suggests the draft rather than volunteering.

Many will be surprised to know that five states require two years of collegiate study for medical licensure and four more, one year. The initiative taken by the University of Buffalo indicates that the Empire State is not in this number. Nor can even Massachusetts claim the honor. In fact, only one eastern state is included—Connecticut, one year, beginning with the medical class of 1914. North Dakota and Iowa led the movement, with a two-year requirement, affecting the present graduating class, at its matriculation four years ago. Eastern culture must follow these states, South Dakota, Minnesota, Colorado, Kansas, Indiana and Utah.

For the country at large, just about one medical graduate in six, in the present year's classes, held a baccalaureate degree. The proportion does not by any means correspond to the state requirement of a partial collegiate course and reaches its maximum, over 50 per cent., in Massachusetts on account of the high requirement of Harvard and the relative lack of competing, low grade colleges, which counteract the tendency of Johns Hopkins in Maryland.

The salutary effects of greater stringency in medical standards include an economic feature of the utmost importance. In 1904, the number of medical graduates reached its maximum,

5,747 and there was good reason to fear that the medical profession would not only suffer but become demoralised by the excess of supply over demand. The total has now fallen to 4,273, less than in 1890 and can not fail to fall still lower within the next few years, however great the matriculation may be. The ancient blessing upon the man who made two blades of grass grow where one grew before, should be doubled upon those who are working to produce exactly the reverse condition for the medical profession. A little more concerted action, producing the same steady diminution of medical students that has occurred since 1904, and we shall reach the point where there will be a vacancy by death, in our ranks, for every new recruit, and an average clinetele increasing by a couple of families a year by the growth of the aggregate population. Even this will not compensate for the decreasing incidence of disease. Let the profession strike while the iron is hot and hasten the time of economic equilibrium.

All of which is written with due acknowledgement to the educational number of the *Journal of the American Medical Association* of August 19, 1911. The annual statistics compiled in these reports represent an enormous amount of work, they are by no means dry reading and the obvious morals illustrate the advantages of a strong professional organisation. One who has followed these reports from year to year can note an almost mathematic correspondence between statistics and practical results. The finger of fate points clearly toward many schools in the present list as doomed to close a worse than useless struggle.

